# Morphology dependent nonlinear optical and photocatalytic activity of anisotropic plasmonic silver†

**DOI:** 10.1039/c8ra08893d

**Published:** 2018-12-11

**Authors:** Jeena Thomas, Prakash Periakaruppan, Vinoy Thomas, Jancy John, Mathew S, Titu Thomas, Jasmine Jose, Rejeena I, Mujeeb A

**Affiliations:** Department of Chemistry, Thiagarajar College Madurai-625009 India kmpprakash@gmail.com prakash_chem@tcarts.in; Centre for Functional Materials, Christian College Chengannur – 689122 India; International School of Photonics, Cochin University of Science and Technology Cochin-22 India; Nano Photonics Division, MSM College Kayamkulam-690502 India

## Abstract

Anisotropic nanoparticles are ideal building blocks for a number of functional materials due to their exceptional and anisotropic optical, electronic, magnetic and mechanical properties. In this work we present systematic studies on morphology dependent ultra-sensitive thermal diffusivity and photodegradation capability of anisotropic plasmonic silver for the first time. Hydrogen peroxide centered synthesis was performed to prepare anisotropic silver nanosystems spherical (14 nm), quasi-spherical (17 nm), elliptical (18 m), rods (aspect ratio 2.1), hexagonal (22 nm) and prisms (19 nm). The synthesized nanosystems were characterized using UV-VIS spectroscopy, high resolution transmission electron microscopy (HRTEM) and band gap analysis. A dual beam mode matched thermal lensing method was adopted for evaluating the thermal diffusivity of the anisotropic system. The present anisotropic nanoparticle system exhibited strong morphology based thermal diffusivity. An increase of 140% in the thermal diffusivity value points to the nonlinear optical application potential of the anisotropic systems. Sunlight mediated photodegradation of methylene blue showed a promising increase in the degradation rate for anisotropic systems compared to other similar systems reported in the literature.

## Abstract 2

Anisotropic plasmonic silver for nonlinear optical and photo catalytic applications.

## Graphical abstract

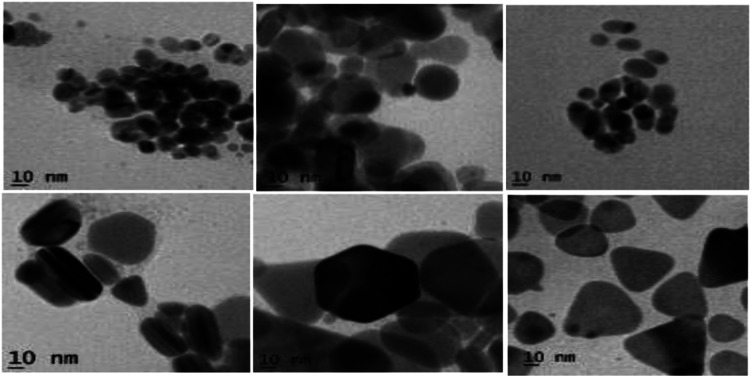

## Introduction

The unique and finely tuned physical and chemical properties of anisotropic nanomaterials have meant that they are gaining attention as a new class of materials for diverse applications.^1^ Since the sphere is the lowest-energy shape, simple reduction of metal salts usually results in the realization of spherical nanoparticles.^2^ It is possible to tune the shape of the nanoparticles by suitably monitoring the experimental parameters like concentration of the metal precursor, reducing agents, stabilizers and reaction conditions such as temperature, time and so on.^1^ The last decade has witnessed systematic efforts to elucidate the depending parameters that directly affect the intrinsic properties of these promising materials. Restricted motion of electrons, holes, excitons, phonons, and plasmons with respect to the physical shape of an object is the main reason for the property changes of the nanosystems. One of the most important changes that is manifested is in colour, because of the confinement of electrons and their changes in electronic energy levels. According to quantum confinement effect, nanomaterials can be categorized into 0D, 1D, 2D and 3D nanomaterials. A large number of anisotropic nanomaterials are reported in the last few years such as nanorods,^3,4^ nanowires,^5,6^ nanotubes,^7^ and so on belonging to 1D and 2D nanomaterials such as triangles,^8,9^ plates and sheets, ribbon,^10–12^ and 3D nanostructures like pyramids,^13,14^ stars,^15^ flowers,^16^ multi-pods,^17–19^ nanourchins,^20^ tadpole,^21^ nano cages,^22^ nanorice,^23^ nanocorns,^24^ nanoboxes,^25,26^ nanocubes^27^ and nano dumb bells.^28^ Alteration of the structural symmetry of nanoparticles can influence their linear optical, non-linear optical, electronic, catalytic and even biological properties.^29–34^ Anisotropic metallic nano particles possess asymmetry axes, and this break in symmetry leads to remarkable optical and chemical properties. Although there have been a large number reports on various anisotropic systems, hydrogen peroxide together with trisodium citrate mediated green synthesis, nonlinear optical and photo catalytic analysis of anisotropic silver nano prisms have seldom been studied. More interestingly a systematic shape dependent study on the above mentioned activities are rarely seen in the literature.

In this context, synthesis and systematic study on nonlinear optical analysis and photo catalytic activity of anisotropic silver nanoparticle systems are deserving research attention, which to our knowledge, has been rarely investigated. Understanding these optical properties of anisotropic systems is a pre-requisite for considering novel nonlinear and catalytic applications. In this paper, we adopted a highly efficient and reproducible mechanism to synthesize anisotropic plasmonic silver systems. We used hydrogen peroxide (H_2_O_2_), an oxidative etchant, as the most essential component, which helps produce anisotropic systems in a facile manner. The purpose of the present study is to prepare anisotropic systems and to study the effect of morphology on the properties such as thermal diffusivity and degradation capability of anisotropic Ag nanoparticles for degrading a pollutant dye, methylene blue. All derived or observed results are compared with similar systems found in the literature.

## Results and discussions

**Fig. 1:**
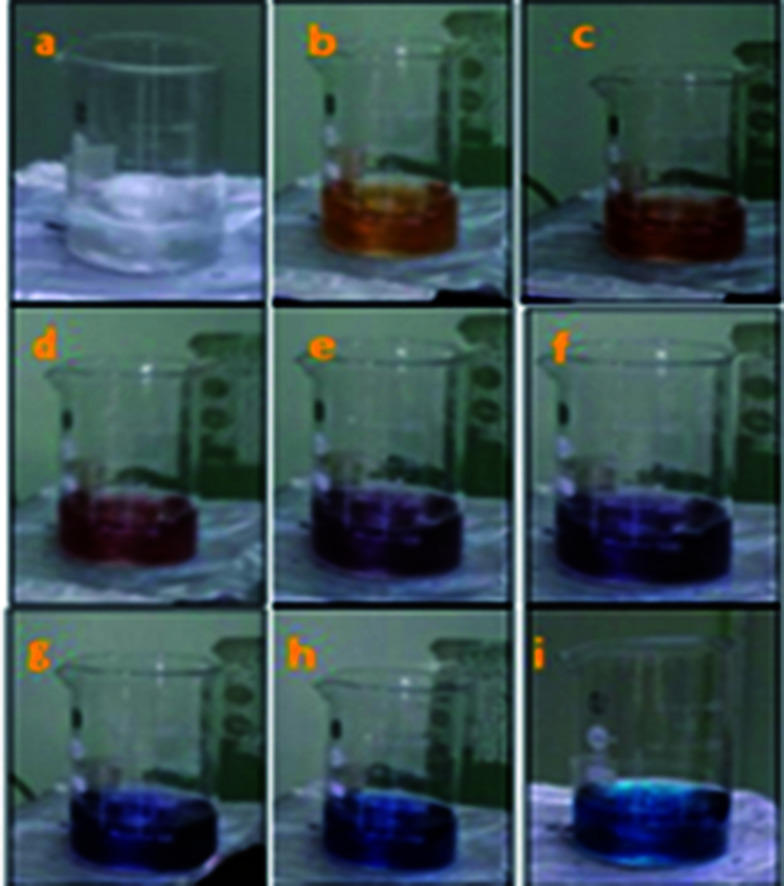
Change from colourless solution to dark blue (a–i) of the synthesized anisotropic Ag NPs.

In the present report, a direct chemical reduction scheme was employed for the synthesis of anisotropic systems. Anisotropic systems of silver are typically prepared by reducing an aqueous solution of silver nitrate (AgNO_3_) with sodium borohydride (NaBH_4_) in the presence of trisodium citrate (TSC). Without the addition of H_2_O_2_, only-spherical silver nanoparticles could be obtained after about 1 min of reaction, as indicated by the colour of the solution (Fig. 1).

**Fig. 2:**
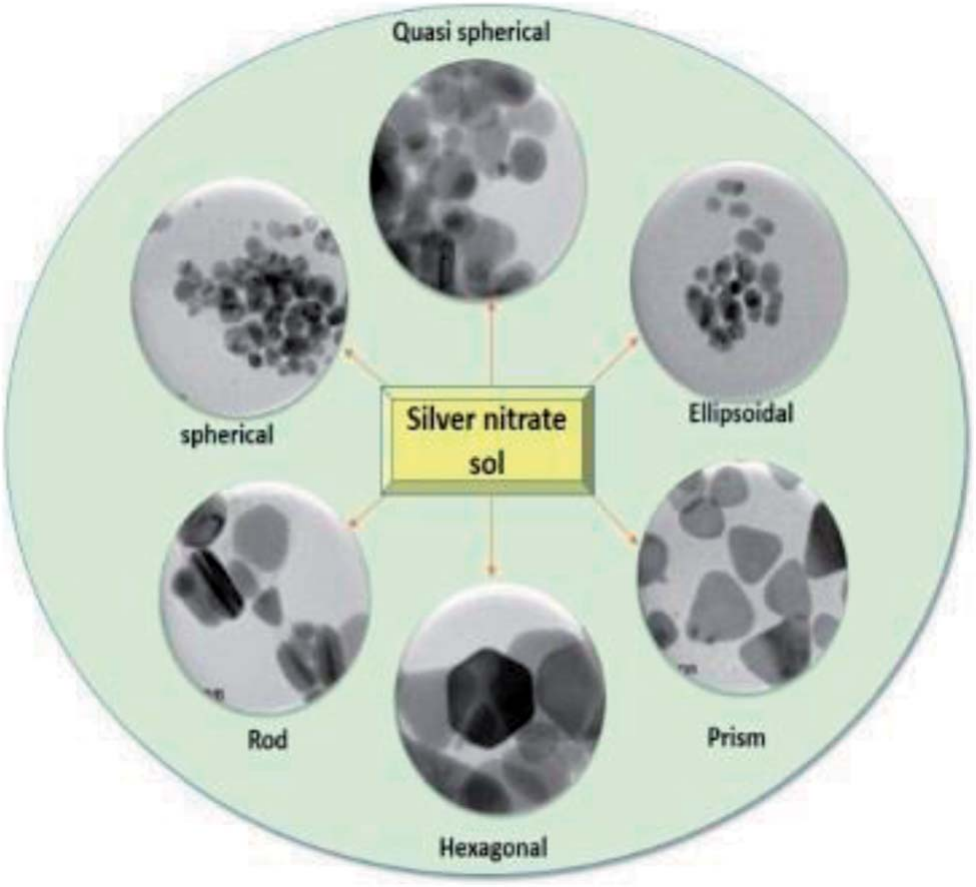
Various shapes of synthesized anisotropic nanoparticles.

**Fig. 3:**
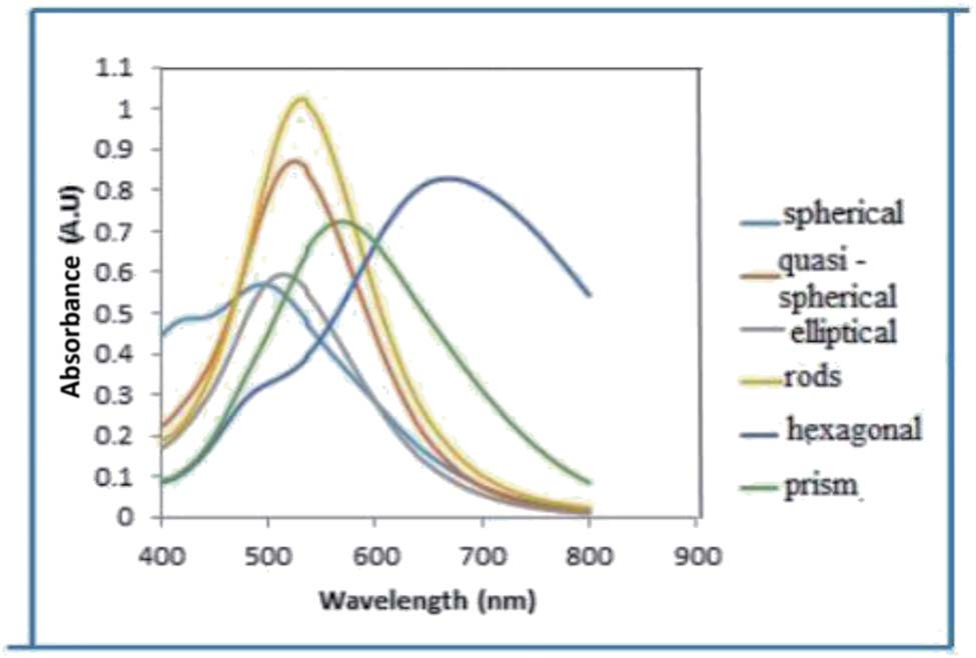
UV-visible spectra of anisotropic AgNPs (concentrations of silver nitrate and trisodium citrate and NaBH_4_ were set as 5 × 10^−2^ M and 75 × 10^−3^ M and 10 × 10^−2^ M respectively. For spherical systems H_2_O_2_ = 0%, quasi spherical-H_2_O_2_ = 1.5 mM, elliptical H_2_O_2_ = 2.0 mM, H_2_O_2_ = 1.5 mM, for rods H_2_O_2_ = 3 mM, for hexagonal H_2_O_2_ = 7.0 mM and for prisms H_2_O_2_ = 15 mM).

The UV-vis absorption spectra of nanofluids containing AgNPs possessing different shapes (Fig. 2) prepared at concentration of 1.5 mM with different particle shapes is shown in Fig. 3. When the concentration of H_2_O_2_ was increased to 2 mM, a sharp peak around 450 nm appeared (Fig. 3), suggesting the formation of both plate-like structures and spherical nano particles, which has also been confirmed by TEM characterization. The sharp characteristic peak of silver nanoparticles at ∼400 nm disappeared as the concentration of H_2_O_2_ was increased to 7 mM. When the concentration of H_2_O_2_ was further increased to 15 mM, the dipole resonance red-shifted to ∼600 nm. H_2_O_2_ is a good oxidizing agent in both acidic and basic condition. The standard potential in the peroxide–water couple (1.763 V (acidic) and 0.867 V (basic)) is higher than that of Ag^+^/Ag (*E*_0_ = 0.7996 V) and hence H_2_O_2_ acts as an effective etchant to dissolve metallic silver. In the present case, H_2_O_2_ acts as an oxidant from the very commencement of the reaction. Upon the injection of NaBH_4_, silver ions are reduced to form silver nanoparticles and the formed nano particles are stabilized by citrate ions. Etching due to H_2_O_2_ inhibits the formation of bigger nano particles. As the result of these competing mechanisms, a dynamic equilibrium is established between the reduction and oxidation processes and hence silver stays as a stable system. Due to the Ag-citrate coordinating interaction and the presence of the powerful etchant, H_2_O_2_, the nuclei are silver nanoparticles containing many defects, favour the growth of anisotropic systems. To investigate the detailed formation process of the silver nano plates, we monitored the change in the SPR peak and evaluated the variation in band gap during growth and nucleation of anisotropic systems. Upon the addition of NaBH_4_, colorless solution became light yellow immediately, suggesting the formation of isotropic silver nano particles. After ∼1 min of reaction, the light yellow solution turned deep yellow in the span of several seconds, suggesting the complete formation of spherical silver nanoparticles. The change in the optical property of the colloidal solution was recorded using a UV/vis spectrometer. Upon addition of H_2_O_2_, colour of the solution then quickly changed from deep yellow to red, green, and finally blue, as evidenced by the Fig. 1. Intensity of the peak of spherical nanoparticles decreased gradually and another peak at ∼500 nm emerged and red-shifted to longer wavelengths, implying the formation and growth of anisotropic systems. The formation and development of anisotropic silver nano systems are completely governed by addition of H_2_O_2_ and the reactions took about 1–3 min. Particle morphology strongly affects the SPR peaks. Particularly for nano rods, one would expect two peaks for (i) a longitudinal plasmon resonance for electron oscillation along rod axis and (ii) a transverse plasmon resonance for electron oscillation along perpendicular direction. Normally the transverse plasmon resonance occurs at about the same position as that of the spherical particles and the longitudinal plasmons shifts towards longer wave lengths.^35^ The separate excitation of these resonances could be possible by using polarized light. In our case, since the rods are not macroscopically oriented (suspended solution) we observed an average over the possible orientations as evidenced in Fig. 3 and in Fig. 6(c).

**Fig. 4:**
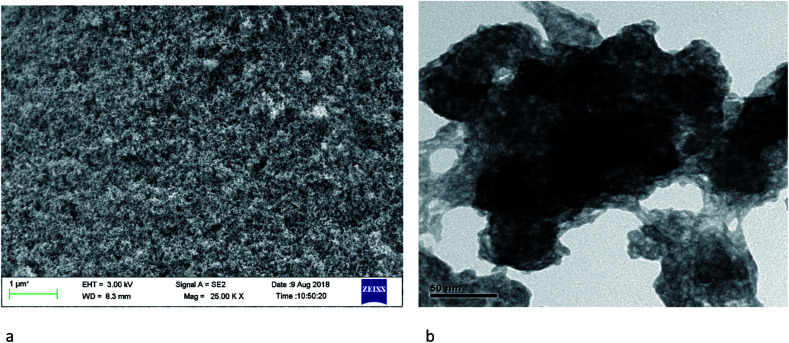
SEM (a) and TEM (b) images of systems in the absence of citric acid and H_2_O_2_.

**Fig. 5:**
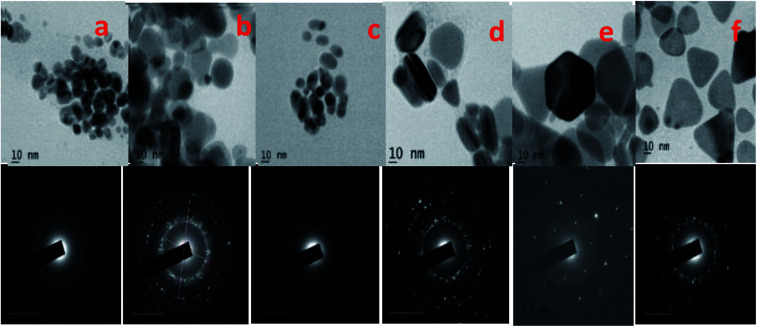
HRTEM images of (a) isotropic, (b) quasi spherical, (c) elliptical, (d) rods, (e) hexagonal and (f) prism and SAED patterns of the respective synthesized anisotropic systems.

The SEM and TEM images of silver nano systems without the addition of citric acid and H_2_O_2_ is shown in Fig. 4. Clear agglomeration of the nano particles could be observed in the system. From the figure it is clear that presence of citrate is essential to get separated and stable nano particles. To eliminate agglomeration, we used a fixed amount of trisodium citrate (2 mL, 75 × 10^−3^ M) in all samples. The HRTEM and respective SAED patterns of the synthesised anisotropic systems in the presence of trisodium citrate, H_2_O_2_ and NaBH_4_ with optimum concentrations are shown in Fig. 5. To evaluate the effect of band gap on the performance of anisotropic systems we evaluated the band gap (*E*_g_) of the anisotropic systems. The *E*_g_ of anisotropic nanoparticles can be determined from the absorption spectra by the Tauc's equation:^36^

**Fig. 6:**
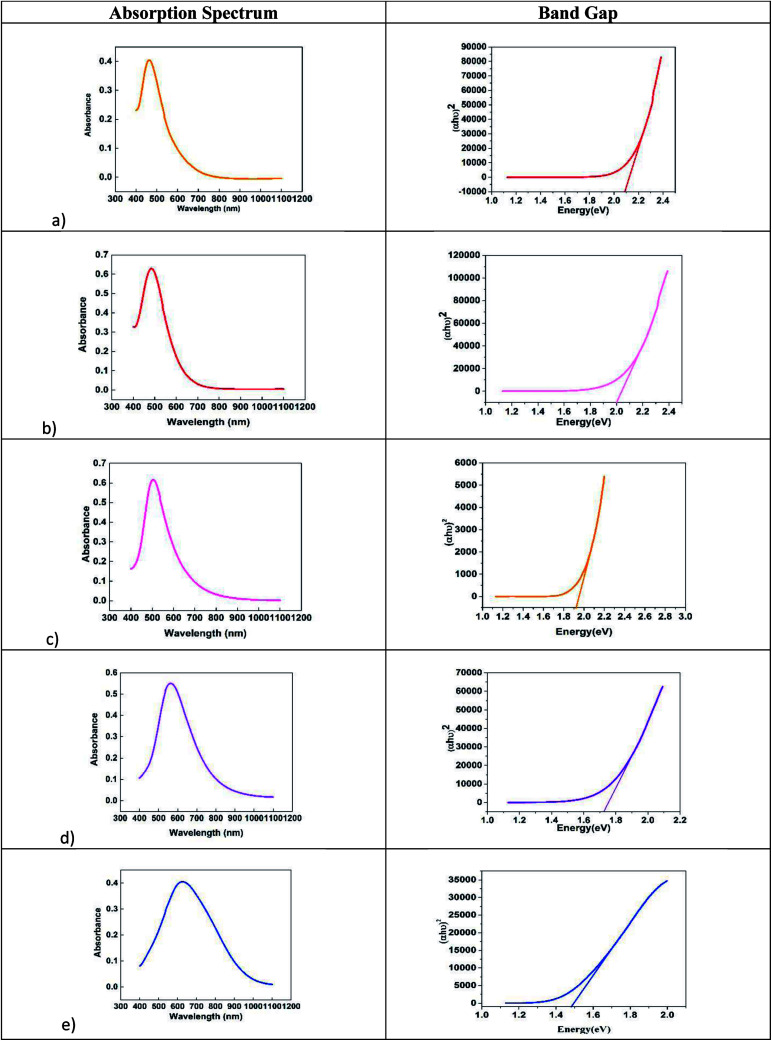
Variation of band gap with morphology of anisotropic systems (a) quasi spherical, (b) elliptical, (c) rods, (d) hexagonal and (e) prisms.

1 (*αhν*)^2^ = *B*(*hν* − *E*_cb_)

here *α* is the absorption coefficient, *hv* is the photon energy, *E*_cb_ is the conduction band energy, and *B* is a constant. According to this relation, by plotting the (*αhν*)^2^*versus* (*hv*) and extrapolation of the linear part of the curve to the energy axis, the conduction band energy of Ag nanoparticles can be determined as shown in Fig. 6. The tabulated energy band gap of the systems are given in Table 1.

**Table 1:** Variation of energy band gap of anisotropic systems with shape

Colour	SPR peak	Morphology	Band gap (eV)
Yellow	425 nm	Spherical	2.4
Orange	465 nm	Quasi spherical	2.3
Red	485 nm	Elliptical	2.0
Pink	505 nm	Rods	1.92
Violet	561 nm	Hexagonal	1.72
Blue	625 nm	Prisms	1.49

### Morphology dependent non-linear optical properties of AgNPs

**Fig. 7:**
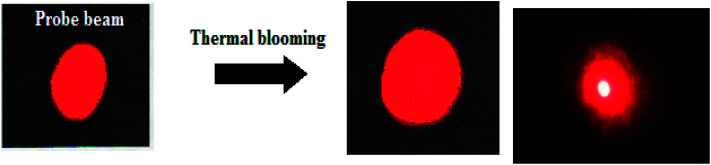
Thermal blooming of anisotropic systems.

To evaluate the effect of morphology of plasmonic particles on the thermo-optic parameter thermal diffusivity, a dual beam mode-matched thermal lens experimental technique was used with a TEM_00_ Gaussian laser beam as the excitation source. A portion of the excitation radiation is absorbed by the sample to jump to the excited states. The excited states lose their energy either energy either radiatively or non-radiatively. During these processes particularly in non-radiative decay processes, heat will be evolved as a consequence of the Stokes' shift, which results in a thermal gradient and hence will lead to a refractive index gradient d*n*/d*t*. The lowest refractive index will be at the centre of the beam path and the radial symmetry of refractive index gradient, makes a diverging lens out of the sample. As the beam pass through the sample blooming will take place (Fig. 7). While blooming, the detector records a loss in intensity corresponding to the temperature change. It is better and convenient to record the time dependent laser beam intensity during the transient heating of the sample instead of measuring the beam spot dimensions.

In a mode matched pump-probe collinear arrangement, measurement of time dependent beam intensity becomes more practical. According to the theory of thermal lensing, the focal length ‘*f*’ of the TL formed in a liquid when a cw laser beam is passed through it at *t* = 0 is given by^37^2
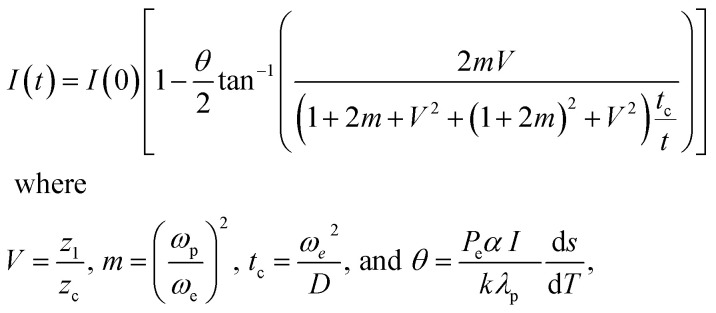
where, *λ*_p_ is the wavelength of the probe beam, *D* and *K* are the thermal diffusivity and thermal conductivity, *P*_e_ is the excitation beam power, *α* is the linear absorption coefficient and *l* is the sample thickness. *z*_c_ = *ω*_0_^2^/*λ* is the confocal distance (cm), *ω*_0_ is the probe beam waist radius, *ω*_p_ and *ω*_e_ are the probe and excitation beam radii at the sample respectively.

**Fig. 8:**
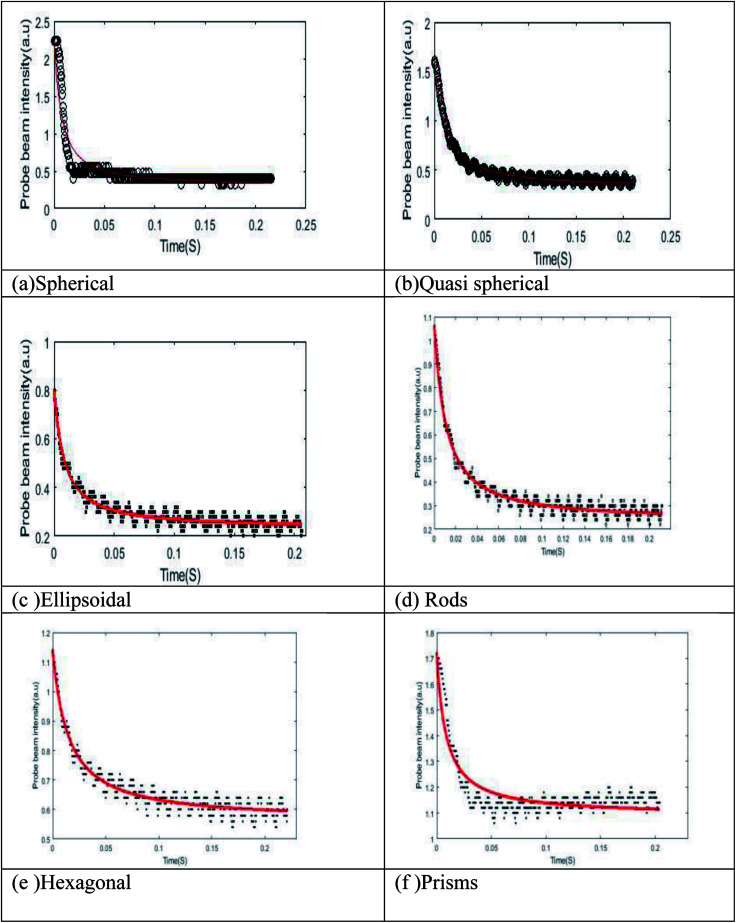
(a–f) Thermal lensing plot of anisotropic nanosystems.

Fig. 8(a)–(f) shows the normalized TL time evolution signal, *I*(*t*)/*I* (0) of the samples. TL setup is quite accurate in measuring this thermal parameter. Similar TL evolution signals were obtained for fluid of other anisotropic silver nanoparticle shapes. Thermal diffusivity parameters *θ*′s, *t*_c_'s, and *D*'s are derived by fitting the theoretical TL signal to the experimentally recorded signal and are given in Table 2.

**Table 2:** Shape dependent nonlinear optical properties of silver

Sample	Theta	*T*_c_	Thermal diffusivity (×10^−8^ m^2^ s^−1^)
*a*	−2.75	0.0528	6.92
*b*	−2.06	0.0693	5.68
*c*	−1.4437	0.0367	9.96
*d*	−1.85	0.0567	6.44
*e*	−0.7529	0.0455	8.03
*f*	−0.4687	0.0223	16.39

From the obtained results, as shown in Fig. 8(a)–(f), it is clear that fluid thermal diffusivity varies with nanoparticle shape. Most of the light (532 nm) absorption and conversion to heat is by silver nanoparticles that produce phonon following the surface plasmon process and thus the particles become heat source. The light absorption and conversion into heat is by transforming from low to high kinetic energy silver ions and citrate molecules but in less efficient way due to small absorbance. A general interpretation for the increase in thermal diffusivity with change of particle shape can be due to phonon scattering at interface or transfer of energy from nanoparticles to liquid. When the particle shape changes, more and more anisotropic, the phonon scattering transfers more energy to the surrounding liquid. From Table 2 it is clear that, for a given concentration of nanoparticles in the fluid, the morphology of the plasmonic particle has a significant influence on the thermal diffusivity parameters. In our case, it is observed that, thermal diffusivity increases with anisotropy of the plasmonic particles. Systems with prism shaped particles are found to be the most efficient carriers of heat energy. In all previous studies it was observed that non-spherical particles exhibit higher thermal diffusivity value than isotropic particles.^38^ To explain the observed phenomena, we could consider a nano fluid containing anisotropic particles as a two component mixture and the mode of heat transport depends upon the way in which particles are distributed.^39^ Both classical and dynamical theories could be adopted to explain the increase in thermal diffusivity values of isotropic systems. Classical theory considers anisotropic nanoparticles as stationary objects in the host fluid. Whereas dynamic models attribute the observed enhancement of thermal diffusivity to many factors such as Brownian-motion-induced micro-mixing, convective heat transfer, lowering of heat resistance of liquid molecules at the particle surface, higher thermal conductivity value of nanoparticles, nanoparticles shape dependent preferred conduction pathways *etc.* In the present case, we believe that the observed increase in thermal diffusivity value could be well explained by using Hamilton–Crosser model.^40^ In this model the calculation of effective thermal diffusivity of a two component heterogeneous mixture involves a shape factor *n* = 3/*ψ* (*ψ*, the sphericity ratio between surface area of the sphere and the surface area of the real particle with equal volumes). According to this model, the effective thermal conductivity of anisotropic nano particle distributed nanofluid can be obtained using the relation wedge3
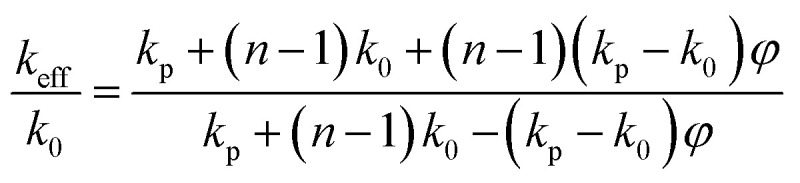
where *k*_0_ is the thermal conductivity of base fluid and *k*_p_ thermal conductivity of nano particles and *φ* is the volume fraction of nanoparticles. Eqn (3) clearly indicates that the thermal diffusivity of all anisotropic particles are larger than that of isotropic nanoparticles. The observed fact is particularly prominent when the thermal discursivity of systems is much higher (>140% times for prisms) than that of host fluid. In short, all anisotropic particles rods, hexagonal, elliptical and quasi spherical shaped particles allow heat transport through the greater length scales and greater surface area of the suspended particles compared to the isotropic counterparts which in turn leads to the observed increase in thermal diffusivity value.

### Morphology dependent photo catalytic activity of AgNPs

**Fig. 9:**
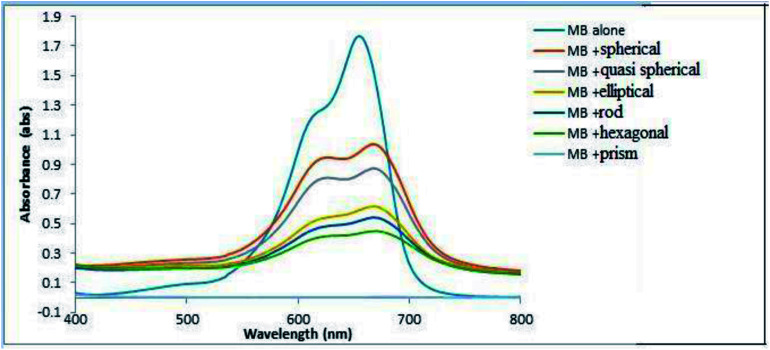
Photo degradation of MB under sunlight with anisotropic particles over a period of 20 minutes.

The role of the metal nanoparticles as an electron transfer catalyst varies with size and shape since its chemistry depends on them.^41^ The shape dependent catalytic activity of silver nanoparticles could be evaluated by employing them on the reduction of a pollutant dye MB. The analysis will be effective and convenient if the study could do with a pollutant/dye possessing a band gap close to the plasmonic particles. Photocatalytic activity of silver nanoparticles on degradation of dye was demonstrated by using the dye methylene blue. MB (3,7-bis (dimethylamino)-phenothiazin-5-ium chloride) is a dark green powder possessing no odour. Traditionally MB is used as dye for painting cotton, wool, and silk. It causes health issues, local burning, nausea vomiting *etc.* It is therefore necessary to eradicate MB dye from the ecosystem. The degradation of methylene blue was carried out in the presence of isotropic and anisotropic silver nanoparticles. In photo catalysis the material absorbs light of energy greater than or equal to its band gap, leading to an excitations of valence band electrons in the conduction band.^42^ Such an excitation leads to the formation of electron–hole pairs and will generate free radicals in the system for redox of the substrate. In aqueous medium MB shows an absorption bands at 664 nm with a shoulder at 614 nm.^43^ The UV-VIS spectrum of the catalytic nature of silver is shown in Fig. 9.

From the UV-visible absorption spectra of methylene blue kept under sunlight for 4–5 hours, it is noted that, the absorbance of the dye decreased by a very small margin. This leads to the understanding that the degradation rate of the dyes under sunlight alone is insignificant (Fig. 8). To improve the photo degradation of these dyes, a small amount of synthesized anisotropic silver nanoparticle solution were added into methylene solution. Fig. 8 shows the temporal evolution of the spectral changes of MB solution mediated by a representative AgNP sample. With increasing illumination time, the absorption peak of MB at 614 nm gradually decreased. Photocatalytic activity of AgNPs originate from the synergy between the SPRs of the Ag nanoparticles and the polarization field. It is very interesting to note that (i) no photolysis of MB is observed in the absence of the photo catalyst under daylight illumination; (ii) a system of isotropic Ag NP alone does not exhibit appreciable photo activity (iii) the observed increase in photo activity of the anisotropic systems may be attributed to the charge transfer and local electric field enhancement.^44^ The solar light was found to be more effective than other irradiation techniques for degrading dyes as reported by the previous studies. During exposure in sunlight, when the photons hit the nanoparticles present in the colloidal mixture, the electrons at the particle surface are excited. The dissolved oxygen molecules in the reacting medium accept the excited electrons from particle surface and are converted into oxygen anion radicals. These radicals break the organic dye into simpler organic molecules leading to the rapid degradation of the dye.^45^ Therefore, the biosynthesized Ag nanoparticles may act as a stable and efficient photo catalyst for degradation of methylene blue under visible light irradiation.

**Fig. 10:**
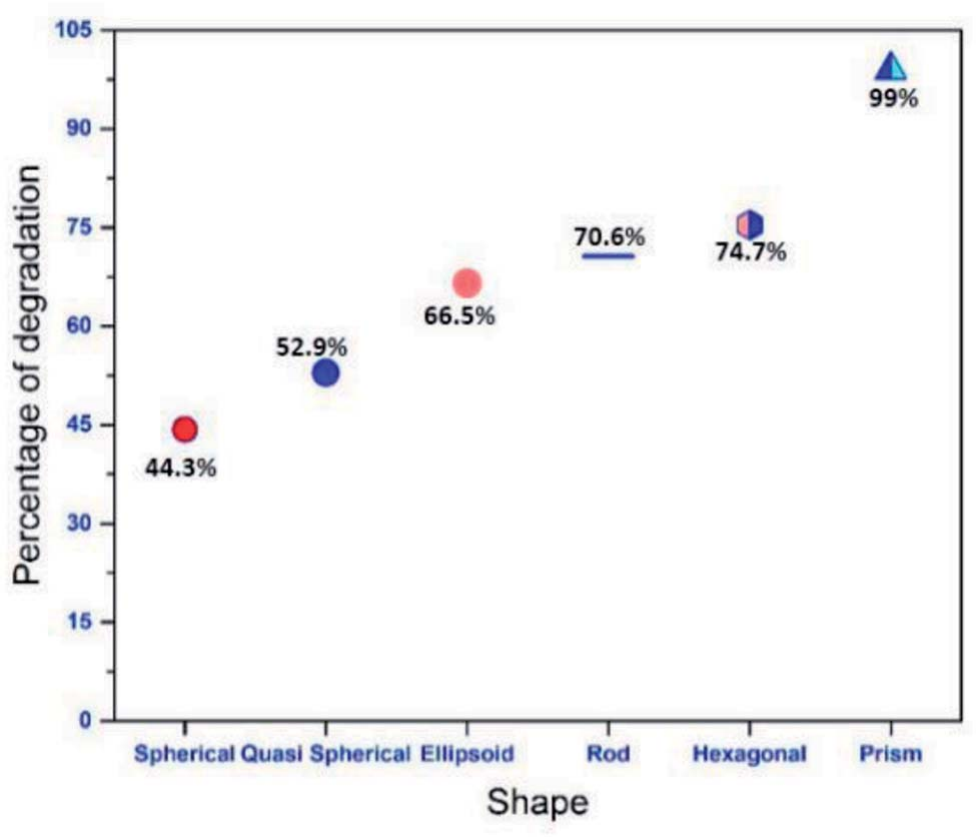
Variation of percentage of degradation with morphology of anisotropic particle for an exposure of 20 minutes.

The crystallographic nature of the nanoparticles along with the morphological structure plays a crucial role in determining the photocatalytic nature of anisotropic AgNPs (Fig. 10). The photocatalytic activity of AgNPs have attributed it to the excitation of the SPR by visible light radiation. The dye forms complex with the capping agent at the surface of the AgNPs which is in turn was photo catalytically degraded.

**Fig. 11:**
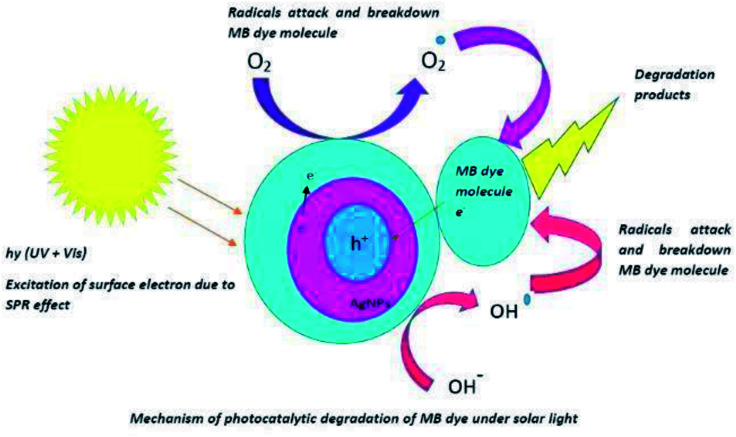
Schematic representation of mechanism of photo-degradation.

AgNPs are known to photo catalytically degrade the organic molecule by absorption of visible as well as UV light of the solar spectrum. When AgNPs absorb visible light of the solar spectra, the surface electrons from the outermost sp band are excited to a higher energy state due to SPR effect. These electrons are readily accepted by the oxygen (O_2_) molecules to form oxygen radicals (O_2_) which attack and degrade the MB dye molecules.^46,47^ Also, the holes generated in the 5sp orbital are filled by the acceptance of electrons from the adsorbed photo-sensitised dye molecule; thereby degrading the MB dye molecule. Absorption of UV irradiation from the solar spectra by the AgNPs causes the excitation of electrons from the 4d orbital to 5sp orbital. This interband transition leads to excitation of many photo generated electrons. These excited electrons interact with the oxygen molecules to form oxygen radicals (O_2_) and the hydroxyl ion to form hydroxyl radical (OH–). Thus formed radicals attack the dye molecule adsorbed onto the surface of the AgNPs and bring about the degradation of the dye. In addition to the degradation of the dyes by the radicals, the holes generated in the d orbital of the AgNPs accept electrons from the adsorbed dye molecule leading to further degradation of the MB dye.^48^ Thus, the anisotropic AgNPs are known for absorption of whole of the light spectrum due to SPR effect and the interband transition of 4d electrons to 5sp band.^49^ This mechanism of photo catalysis by UV and visible light irradiation by AgNPs in the degradation of MB dye is presented in Fig. 11.

**Fig. 12:**
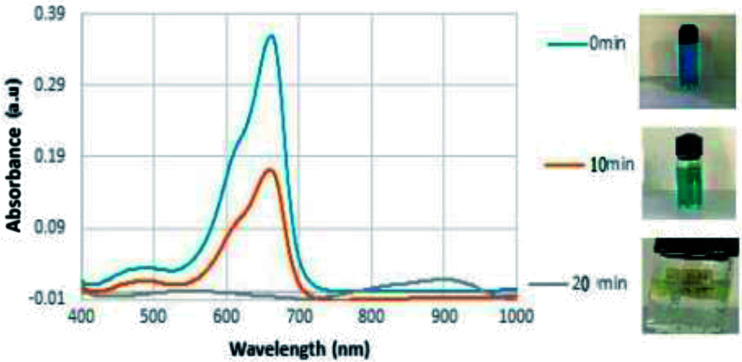
Photo catalytic activity of nanoprisms when exposed to sunlight for a time period of 20 minutes.

The variations of degradation ability of each anisotropic particle for a time duration of 20 minutes is shown in Fig. 10. From the figure it is clear that, as a catalyst, Ag nanoprisms are superior to Ag nanospheres and other anisotropic systems in their outstanding plasmon resonance behaviour, including broader absorption range due to multiple resonances. With broader absorption, nanoprisms were capable of utilizing more photons in the visible light range than nanospheres by either hot electrons formation or resonant energy transfer in further photo catalysis events. It has been observed that conductive electrons tend to concentrate at sharp corners at resonance, which would result in stronger LSPR effects to trigger the photo degradation process; however, no similar phenomenon could be observed for nanospheres with a smooth surface. The photo catalytic activity of the anisotropic prism is superior comparable to any other Ag nanomaterials reported in the literature as indicated in Fig. 12 (a comparison with similar reports systems has been given in the ESI†).

Anisotropic AgNPs exhibited enhanced photocatalytic activity, opening new avenues in the field of photo catalysis rather than the conventional semiconductor nanoparticles. Anisotropic AgNPs absorb the solar radiation and lead to the excitation of electrons on the surface by the surface plasmon effect. The anisotropic AgNPs synthesised in the current study are photo catalytically active under solar light and thus offer sustainable solutions for the treatment of dye contaminated wastewater. These nanoparticles are synthesised without the use of polluting solvents or extreme conditions of temperature and pressure, thus exploring a lower chemical footprint on the environment. These anisotropic AgNPs can be used for large scale treatment of wastewater by utilisation of solar energy, an abundantly available form of renewable energy. The synthesis of these AgNPs and their application as solar light active photo catalyst features energy efficient, eco-friendly and a green technology.^49^

## Experimental

### Synthesis of anisotropic plasmonic systems

In the direct chemical reduction scheme, anisotropic silver nanoparticles were successfully prepared at room temperature. The formation of anisotropic silver nanoparticles (AgNPs) was obtained by the reduction of silver ion to silver from silver nitrate by a reducing agent sodium borohydride. This shape formation was controlled by trisodium citrate (TSC) and hydrogen peroxide (H_2_O_2_). In our synthesis, the concentrations of silver nitrate, trisodium citrate and NaBH_4_ were set as 5 × 10^−2^ M, 75 × 10^−3^ M and 10 × 10^−2^ M respectively. Normally, 98 mL aqueous solution (starting solution) combining silver nitrate (0.2 mL) and tri sodium citrate (2 mL) was continuously stirred at room temperature in air. NaBH_4_ (2 mL) was rapidly injected into the mixture to start the reduction process, suddenly leading to a light yellow solution. It was noticed that the colour change of the solution stops within two minutes indicating the completion of reduction of silver nitrate by NaBH_4_. In the absence of H_2_O_2_, the solution retained its pale yellow colour. Different amounts of H_2_O_2_ (30%) solution was added to obtain anisotropic structures. Upon addition of H_2_O_2_ to the starting solution, colour of the solution changed from deep yellow to red, green, and finally blue, as evidenced in Fig. 1. Fig. 1 shows the colour change of the prepared anisotropic solutions. The formation of anisotropic Ag NPs was preliminarily characterized by visual observation of the change in colour of the solution. UV-visible absorption spectra were recorded in the range 300–900 nm using a Jasco (V-560) model double beam spectrophotometer. The size and morphology of Ag NPs were examined by transmission electron microscopy (JEOL, JEM 2100 model instrument).

### Thermal lensing – experimental

Ultra-sensitive dual beam mode matched TL technique was used to measure non radiative decay and thermal diffusivity. A 532 nm diode pumped solid state laser (DPSS) with a maximum power of 150 mW was used as the excitation source and a 2 mW He–Ne laser used as the probe. The two beams were focused in to the sample cell such that the beam area at the sample plane was the same for both pump and probe resulting in a mode matched TL configuration. Attenuators were used for adjusting the power at the sample to avoid aberrations.

### Photo catalytic activity

In the current study, the photocatalytic activity of the synthesised AgNPs was tested in terms of its efficacy to photo catalytically degrade MB dye. Photocatalytic degradation of MB dye at a concentration of 50 mg L^−1^ was carried out in 100 mL of aqueous solution using 2 mg of the synthesised AgNPs. The reaction mixture was continuously stirred using magnetic stirrer under bright solar irradiation. To test for any possible solar photolytic reaction, a control batch experiment was conducted under sunlight in the absence of AgNPs by maintaining the similar conditions as that of photocatalytic degradation experiments. An experiment was also conducted in the presence of AgNPs but under dark conditions, to test for any possible adsorption of the dye. Samples were withdrawn from the reaction mixtures at regular intervals of time and the dye concentration was determined through absorbance measurement at a wavelength of 614 nm using UV–vis spectrophotometer and the precalibrated data. Percentage dye removal or degradation was calculated using the relation^50,51^4
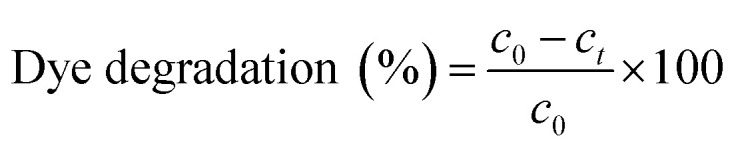
where *C*_0_ is the initial dye concentration and *C* is the dye concentration at any time during the reaction.

## Conclusions

In this paper, we report a simple green method for the preparation of different shaped (quasi spherical, elliptical, rods, hexagonal and prisms) silver nanostructures based on hydrogen peroxide and tri sodium citrate. A systematic study showed that hydrogen peroxide plays the crucial role in determining the morphology of the nano systems. Thermal diffusivity measurements through ultrasensitive dual beam mode matched thermal lensing and sunlight mediated photo catalysis of methylene blue dye revealed that anisotropic nano structures offer much increased value for thermal diffusivity and photo degradation rate. Prism shaped nano systems showed much higher thermal diffusivity and dye degradation rate than other systems reported in the literature. The synthesized colloids were stable for long span shelf life which increases the possibility of applications.

## Conflicts of interest

The authors declare that there is no conflict of interest regarding the publication of this paper.

## Supplementary Material

RA-008-C8RA08893D-s001
