# Supplementary material for: Morphology dependent nonlinear optical and photocatalytic activity of anisotropic plasmonic silver
Source: RSC Adv. 2018 Dec 11;8(72):41288–98. doi: 10.1039/c8ra08893d (PMC9091622; doi:10.1039/c8ra08893d)
Supplement: RA-008-C8RA08893D-s001 [file RA-008-C8RA08893D-s001.pdf]

This journal is © The Royal Society of Chemistry 2018

A comparison of photo degradation Capability of Anisotropic systems with similar systems found in Literature

Catalyst/ concentration	Shape	Dye	Degradation time (Min.)	References
AgNPs/30 mL mL^{-1}	Spherical	MB	9	1
AgNPs	Spherical	MB	20	2
AgNPs	Spherical	MB	12	3
AgNPs/0.03 mg mL^{-1}	Spherical	MB	5.5	4
AgNPs	Spherical	MB	3	5
AgNPs/10–100 mg mL^{-1}	Spherical	MB	8	6
AgNPs/79.78 mL	Spherical	MB	60	7
Fe_3O_4@PDA–Ag 5 mg mL^{-1}	Spherical	MB	22	8
Fe_3O_4@Ag/1.6 mg mL^{-1}	Core shell	MB	6	9
Ag–Fe_3O_4@ C core–shell NCs/-	Spherical	MB	6	10
Ag-NSs/0.001 mg mL^{-1}	Nano shell	MB	100(s)	11
Silver (1mM/25ml)	Nano sphere colloid	MB	480	Present work
	Quasi spherical		320	
	Elliptical		280	
	Rod		220	
	Hexagonal		110	
	Prism		20	

References

1. Sinha, T, and Ahmaruzzaman, M. *J. Colloid. Inter. Sci.* 2015, **453,** 115-131.
2. Suvith, V. S., and Daizy Philip. *Spectrochimic. Acta* 2014, **118**, 526-532.
3. Rajan, A, Vidya V, and Daizy P. *J. Mol. Liquids* 2015, **207**, 231-236.
4. Joseph, S, and Beena M. *J. Mol. Liquids* 2015, **204**, 184-191.
5. Bogireddy, N. Kumar R, Hoskote A, Kiran K, and Badal K, Mandal. *J. Environ. Chem. Engg.* 2016, **4(1)**, 56-64.
6. Vidhu, V. K., and Daizy Philip. *Spectrochim. Acta Part A: Molecular and Biomolecular Spectroscopy,* 2014, **117**,102-108.
7. Wunder, S, Frank P, Yan L, Yu M, and Matthias B, *J. Phys. Chem. C* 2010, **114(19),** 8814-8820.
8. Xie Y, Bin Y, Haolan X, Jian C, Qingxia L, Yonghong D, and Hongbo Z. *ACS. Appl. Mater. Interfaces*. 2014, **6(11)**, 8845-8852.
9. Sharma, G, and Pethaiyan J. *Eur J. Inorg. Chem*. 2013, **36,** 6126-6136.
10. Zhu M, Chengjiao W, Dehai M, and Guowang D. *J. Mater. Chem. A* 2013, **1(6)** 2118-2125.

11. Vellaichamy Balakumar and Prakash Periakaruppan. *RSC Adv*. 2016,. **6(38)** 31653-31660
